# Cavity dynamics after the injection of a microfluidic jet onto capillary bridges†

**DOI:** 10.1039/d2sm01285e

**Published:** 2022-12-01

**Authors:** Miguel A. Quetzeri-Santiago, David Fernandez Rivas

**Affiliations:** a Mesoscale Chemical Systems Group, MESA+ Institute and Faculty of Science and Technology, University of Twente P.O. Box 217 7500AE Enschede The Netherlands m.a.quetzerisantiago@utwente.nl d.fernandezrivas@utwente.nl

## Abstract

The ballistics of solid and liquid objects (projectiles) impacting on liquids and soft solids (targets) generally results in the creation and expansion of an air cavity inside the impacted object. The dynamics of cavity expansion and collapse depends on the projectile inertia as well as on the target properties. In this paper we study the impact of microfluidic jets generated by thermocavitation processes on a capillary bridge between two parallel planar walls. Different capillary bridge types were studied, Newtonian liquids, viscoelastic liquids and agarose gels. Thus, we compare the cavity formation and collapse between a wide range of material properties. Moreover, we model the critical impact velocity of a jet traversing a capillary bridge type. For agarose gels with a storage modulus of 176 Pa, the critical velocity is well predicted by the model used for liquids. However, the predicted critical velocity for liquids deviates for agarose gels with a storage modulus of 536 Pa and 3961 Pa. Additionally, we show different types of cavity collapse, depending on the Weber number and the capillary bridge properties. We conclude that the type of collapse determines the number and size of entrained bubbles. Furthermore, we study the effects of wettability on the adhesion forces and contact line dissipation. We also conclude that upon cavity collapse, for hydrophobic walls a Worthington jet is energetically favourable. In contrast, for hydrophilic walls, the contact line dissipation is in the same order of magnitude of the energy of the impacted jet, suppressing the Worthington jet formation. Our results provide strategies for preventing bubble entrapment and give an estimation of the cavity dynamics, of relevance for, among others, needle-free injection applications.

## Abstract 2

The ballistics of a microfluidic jet impacting on a capillary bridge. High speed imaging and image analysis was used to track the cavity profiles in detail, unravelling the impact phenomena for each material.

## Graphical abstract

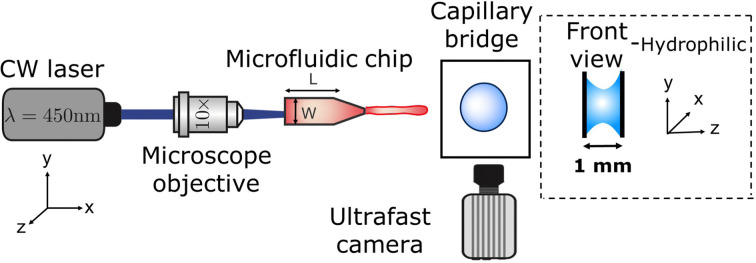

## 1. Introduction

The impact of droplets and jets on other liquids and solid objects is a recurrent ballistic phenomenon, both in nature and in industrial and medical contexts.^1–3^ Rain droplets impact bodies of water, leaves or soil;^1,4,5^ droplets for inkjet printing and additive manufacturing impact previously deposited liquid layers or dry paper;^6,7^ and in needle-free injections a liquid jet is directed to impact and penetrate the skin.^8^ Droplet impacts onto pools have been studied since the works of Arthur Mason Worthington at the start of the 20th century.^9^ With the development of high speed cameras more features of these phenomena have been discovered and disentangled. However, the input parameters, the outcomes, and practical applications are so diverse that it is still an active research topic.^10–13^

Previous research on water entry of liquid and spheres has focused on the critical energy necessary for air entrainment into the pool, the collapse of the entrained air cavity and the subsequent formation of a liquid jet that travels in the opposite direction of impact, *i.e.*, a Worthington jet.^14–16^ The time of collapse, cavity geometry and Worthington jet depend on both the properties of the liquid pool and the impacting object.^9,17–21^ The phenomenon is usually well described by the ratio between the surface tension, inertia and hydrostatics. Regime maps are often constructed in terms of the Froude number Fr = *U*_0_^2^/(*gD*_0_) and the Weber number We = *ρ*_0_*D*_0_*U*_0_^2^/*γ*_cb_, where *ρ*_0_ and *D*_0_ are the density and diameter of the impacting object, *U*_0_ is the impact velocity, *γ*_cb_ is the surface tension of the impacted object and *g* is the acceleration due to gravity.^22^ Recently, research has been extended to the impact on non-Newtonian liquids as well as soft solids, such as hydrogels.^23,24^ The results on hydrogels show the resemblance of the cavities, shape, and closure to those formed in water, albeit, for hydrogels, elasticity needs to be considered. An effective parameter to introduce the elastic properties on the collapse of the cavity is the elastic Froude number Fr_e_ = *ρU*_0_^2^/*G*, where *G* is the shear modulus.^24^

Past studies have mainly explored the impact onto semi-infinite pools and the proximity effects of solid interfaces remain relatively unexplored.^25–27^ Zou *et al.* in 2013 studied the impact of liquid droplets on pools contained in tubes with different diameters.^26^ The study concluded that the cavity collapse and entrainment of a large bubble was dependent on the Weber number and the distance from the surrounding walls to the impact point.^26^ Furthermore, the impact of a micrometer sized projectile at Fr ≫ 1 and We ≫ 1, where the cavity collapse is driven by the capillary forces instead of the hydrostatic pressure, has not been thoroughly described. This impact regime is important in processes such as 3D printing, spray painting and needle-free injections, as the relevant length scale is in micrometers and gravity does not influence these kinds of processes.^8,28–30^

Here, we study the impact of microfluidic jets onto millimetre sized droplets and agarose gels confined between two glass slides, *i.e.*, capillary bridges. From a perpendicular view of the impact, we can compare the cavity formation and collapse between a wide range of material properties. We study the formation and collapse of the air cavity upon impact and extract the cavity profiles for each frame. Furthermore, by changing the wettability of the walls we study the influence of the liquid–gas interface curvature and the contact line forces on the impact outcome. These experiments give us valuable information of jet interaction with materials ranging from liquids (Newtonian and viscoelastic) to soft solids (agarose gels). With Newtonian liquids we can vary the viscosity and surface tension, while for non-Newtonian liquids we can vary their relaxation time and thus their elastic response. Similarly, with agarose gels we can control the storage modulus. Furthermore, agarose gels are commonly used as skin surrogates in experiments because of their ease of preparation, resemblance to biological tissue at a macroscopic level, and transparency. These gels do not have the complexity of skin, as skin is heterogeneous and has multiple layers, each with different properties. However, agarose gels are widely used.^31,32^

The impact conditions we describe in this study, namely the confinement of the liquid and agarose gels, have relevance for needle-free injections. Our group has highlighted this elsewhere.^8,32^ In particular, the knowledge about the impacts of nanoliter jets (≈10 nL) is insufficient and much less studied than conventional jet injectors (1 mL). In most reported studies, either the skin or surrogate is flat. Yet, we aimed at expanding the knowledge about situations where the topography of the target (including, skin, but also coatings and other relevant materials) might be curved. In particular, in needle-free injections, the topography of skin might be curved and confinement can occur due to the skin topography and wrinkles.^33–35^ In this study we employ a jet impact velocity *U*_0_ and jet diameter *D*_0_ of [8–69.5] m s^−1^ and [50–120] μm, respectively. This velocity range is lower than that used in several needle-free injectors which are between 100 and 200 m s^−1^.^36–38^ However, commercial jet injectors are not as collimated as the jets generated through thermocavitation, and the volume range differs by six orders of magnitude.^37,39^ Interestingly, a jet with a diameter of 100 μm impacting on skin at ≈40 m s^−1^ can make an injection in *ex vivo* experiments.^40^ The exact mechanism of this injection has not yet been clarified due to the limitations posed by skin properties, namely its opaqueness. Thus, in this paper we explore the dynamics of the jet impact in this “low” velocity injection regime in transparent materials with a different storage modulus to resemble real skin.

## 2. Experimental methods

To create capillary bridges, liquid or agarose gel (before curing) droplets were confined between two Borofloat glass slides standing 1 mm apart. This separation was chosen as experiments for injecting in agarose gels are carried out for similar confinement.^31,39,40^ Wrinkles in skin can provide similar confinement to the injection site.^33^ Droplets of 15 and 10 μl were generated with an Eppendorf pipette and placed in between the opening of the glass slides. In this way the droplet was driven by capillary forces until it adopted a circular shape as described in ref. 41. The liquids used partially wet glass with a contact angle of 23 degrees. Capillary forces maintained the droplet pinned once equilibrium was reached. Experiments were also conducted in capillary bridges between hydrophobic walls. In this case, the glass walls where coated with Glaco spray coating. For the latter, the coated glass walls were positioned parallel to the *xz* plane (rather than parallel to the *xy* plane), as the capillary bridge would slide otherwise. The camera was positioned parallel to the *y* axis. The contact radius *R*_c_ between the capillary bridge and the glass was ≈3 mm, *i.e.*, comparable to the capillary length. Hence, we expect no influence of gravity on the capillary bridge geometry. Lastly, no difference was observed between placing the cross section of the capillary bridge parallel to the *xz* plane or perpendicular to it.

The capillary bridges were formed with three distinct types of materials; Newtonian liquids with different viscosities, viscoelastic solutions with different relaxation times *λ*, and agarose gels with different storage moduli *G*. The Newtonian liquids used were water and an aqueous glycerol solution at 78 wt%. The viscoelastic liquids were water based polyethylene-oxide (PEO) solutions with molecular weights ranging from 600 kDa to 1000 kDa at different concentrations. Agarose gels were prepared by diluting agarose powder (OmniPur agarose, CAS No. 9012-36-6) in Milli-Q water at different concentrations and heated up for 45 s in a microwave at 700 W. The solutions were cooled down until they reached 50 degrees Celsius before casting between the glass slides. All chemical compounds were acquired from Sigma-Aldrich.

We impacted the capillary bridges with microfluidic jets generated through thermocavitation with velocities, and diameters in the range of *U*_0_= [8–69.5] m s^−1^ and *D*_0_= [50–120] μm, respectively. This range of *U*_0_ and *D*_0_ is relevant for needle-free injections as a jet with a diameter of 100 μm impacting on skin at ≈40 m s^−1^ can make an injection in *ex vivo* experiments.^40^ Thermocavitation is a process where liquid is vaporised and generates an expanding bubble.^42,43^ This expanding bubble subsequently pushes the liquid in front of it, creating a liquid jet.^44,45^

**Fig. 1:**
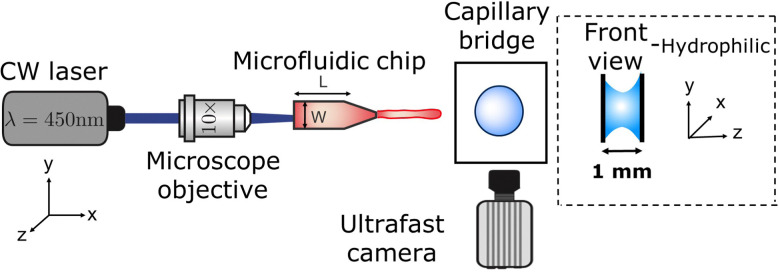
Schematic of the experimental setup. A microfluidic jet is generated by a thermocavitation process and is directed to impact a liquid on a droplet or agarose droplet placed in between two glass slides separated by 1 mm, forming a capillary bridge. The process is recorded *via* shadowgraph imaging with a high-speed camera.

The experimental setup, as shown in Fig. 1, consists of a Borofloat glass microfluidic chip, which acts as a reservoir for the liquid and controls the jet ballistics.^45^ The liquid used for the jet is a water solution containing a red dye (Direct Red 81, CAS No. 2610-11-9) at 0.5 wt%. The red dye enhances the laser energy absorption from a continuous wave laser *λ* = 450 nm (Roithner LaserTechnik, nominal power of 3.5 W). The laser is focused at the microfluidic chip with a 10× objective. The liquid jet impacting speed *U*_0_ and its diameter *D*_0_ were controlled with the microchannel geometry, its distance to the focal point of the microscope objective and by varying the laser power from 0.4 W to 2.1 W. A detailed description of the system can be found elsewhere.^46^

The surface tension of all the liquids was measured with the Pendent Drop ImageJ plugin.^47^ The surface tension *γ* and shear viscosity *μ* of the agarose gels was obtained through the following linear relationships,^48^

1 *γ* = 0.001022*G* + 0.07229

2 *μ* = 6.005 × 10^−5^*G* + 0.00834

The rheological properties of all the materials were measured with an Anton Paar MCR 502 rheometer. The properties of the liquids and agarose gels are reported in Table 1. The shear viscosity (*μ*) of the different capillary bridges spans two orders of magnitude, while their surface tension (*γ*) spans three orders of magnitude.

**Table 1:** List of fluids and agarose capillary bridge used providing their shear viscosity *μ*, surface tension *γ* and density *ρ*. The viscoelastic relaxation time *λ* is also shown for the polyethylene-oxide solutions and the storage modulus *G* for the agarose gels

Fluid/Gel	*μ* (mPa s)	*γ* (mN m^−1^)	*ρ* (kg m^−3^)	*λ* (ms)	*G* (Pa)
Water	1.0	72.1	998	—	—
Glycerol 78 wt%	43.60	65.2	1212	—	—
Water & red dye 0.5 wt%	0.91	47.0	1000	—	—
PEO 600k 0.1 wt%	1.56	63.1	996	0.31 ± 0.04	—
PEO 600k 1.0 wt%	21.70	62.9	998	1.32 ± 0.08	—
PEO 1 M 1.0 wt%	44.74	59.2	998	6.14 ± 0.69	—
Agarose 0.15 wt%	18.96	252.9	1000	—	176 ± 29
Agarose 0.25 wt%	40.53	620.2	1000	—	536 ± 21
Agarose 0.50 wt%	256.34	4120.4	1000	—	3961 ± 722

The processes of bubble generation, jet ejection and impact on the capillary bridges were recorded with a Photron Fastcam SAX coupled with a 2× microscope objective. A typical experiment duration was ∼5 ms and the camera resolution was set to 768 × 328 pixels^2^ at a sample rate of 50k frames per second with an exposure time of 2.5 μs. Image analysis to extract the jet diameter, impact velocity and cavity dynamics was performed with a custom generated MATLAB script.

## 3. Results and discussion

In this section we describe liquid jet impacts on liquid and agarose capillary bridges, and compare both cases with previous results of jets impacting liquid pools and droplets.

In all experiments, upon jet impact on the capillary bridge, an air cavity is created. The cavity continues expanding in the *x* direction with a velocity *U*_c_ proportional to the impact speed *U*_0_. Furthermore, due the entrained air and momentum conservation, this cavity also expands in the radial direction.^11^ At a certain instant, the cavity stops expanding, reaches a maximum volume and starts retracting or pinches off. In this process, trapping of air bubbles inside the capillary bridge can occur depending on the impact and liquid characteristics. We then extract the cavity profile and the cavity front position for each video frame.

In the following sections we discuss the cavity dynamics. In Section 3.1, we focus on the expansion of the cavity upon impact and we provide two models and compare with experimental results. Furthermore, we study the critical transition Weber number for the jet to fully traverse or get embedded into the capillary bridge, and compare it to jets impacting on pendant droplets. In Section 3.2, we discuss the different types of cavity collapse in terms of the liquid and impact characteristics. Finally, in Section 3.3 we study the effect of wettability on the cavity formation and collapse based on the energy dissipation on the contact line.

### 3.1. Model for the cavity expansion

When a capillary bridge is impacted by a jet, a cavity is generated. The cavity keeps expanding until it reaches a maximum depth. If the maximum depth of the cavity is larger than the capillary bridge diameter *D*_cb_, the jet will traverse the capillary bridge. In our case, the cavity radial expansion is hindered by the walls confinement, depending on the impact velocity. For liquids, a cavity forms even for the smallest jet impact velocity. In contrast, for the agarose gels a critical impact velocity is necessary to generate a cavity, *i.e.*, inertia has to overcome the elasticity of the agarose gel. This critical velocity depends on the Young's modulus of the gel and polymer content.^36,49^

Multiples studies have been dedicated to the cavity expansion and the evolution of its dimensions after a water entry event.^12,46,50–52^ At the moment of jet impact, the cavity creation is dominated by inertia (We ≫ 1). By considering the jet inertia in the x direction and that at the beginning of the crater growth d*H*_c_/d*t* = *U*_c_ ≈ *U*_0_/2, where *U*_c_ is the velocity of the tip of the expanding cavity *H*_c_. Therefore *H*_c_ is expected to grow linearly with time and the jet inertia is predominantly converted to the momentum of the surrounding liquid.^50^ By solving the two dimensional Rayleigh equations in cylindrical coordinates,3
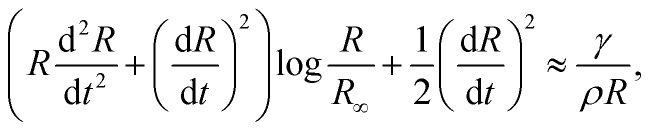
we can predict the evolution of the cavity radius *R*(*x*,*t*). Since We ≫ 1 and Re ≫ 1, surface tension can be neglected during expansion and *R*(*t*) ≈ (*t* − *t*_0_)^1/2^, where *t* = *H*_c_/*U*_c_ and *t*_0_ = *x*/*H*_c_, and the approximate cavity profile is,^50^4
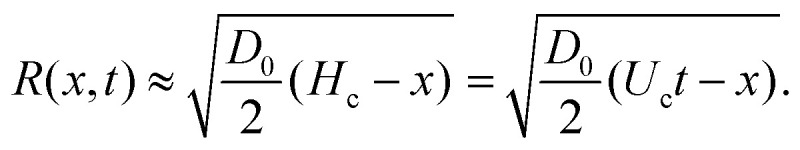


**Fig. 2:**
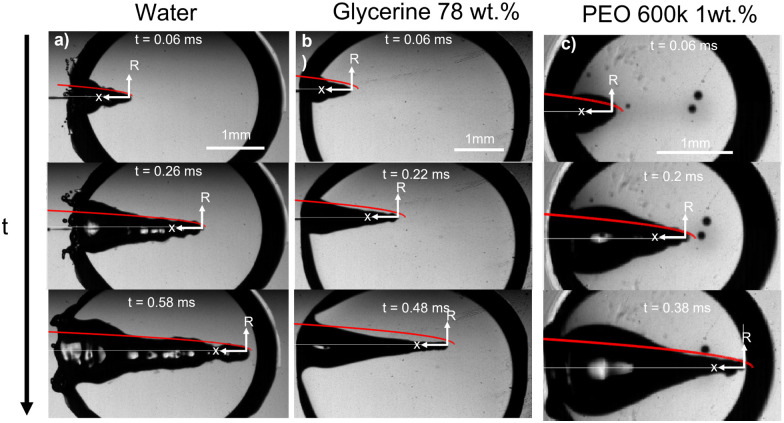
Experimental images showing a liquid jet impacting (a) a water capillary bridge, We = 495 and *D*_0_= 93 μm (Movie 1 in the ESI†), (b) a glycerol 78% capillary bridge We = 483 and *D*_0_= 74 μm (Movie 2 in the ESI†) and (c) a PEO 600k 1 wt% capillary bridge We = 498 and *D*_0_= 80 μm (Movie 3 in the ESI†). In all the images a comparison with the theoretical profile given by eqn (4), is shown as a red line. We adapted the code from ref. 34, where instead of solving for a train of droplets we used eqn (4) to predict the cavity radius. This code evaluates eqn (4), at a given time and plots it on top of the experimental images to calculate the depth we use *H*_c_ = *U*_c_*t*. The videos associated with these figures can be found in the ESI.†

Fig. 2 shows experimental images of the expanding cavity created after the impact of a microfluidic jet on a capillary bridge of different liquids. This figure also compares the experimental profile with the theoretical profile from eqn 4 (shown as a red line). The theory considers that the jet has a uniform radius across its length, therefore a perfectly parabolic profile is expected. However, as the jets in our experiments have a bigger head than the following liquid, and it breaks up before the collapse of the cavity, the cavity radius at the impact point (*R*(0,*t*)) is underpredicted for all cases.

Fig. 2a, shows the comparison between the theoretical profile and experimental profile after the impact on a water capillary bridge. The theoretical profile fits the shape of the cavity generated in water, albeit, the cavity is not as smooth as theory predicts. This difference arises from assuming a perfectly cylindrical and steady jet. In reality the jet breaks up and subsequent droplets impact the base of the cavity generating surface waves across all the cavity^11^ (see Movie 1 in the ESI†).

For the glycerol 78 wt% capillary bridge we observe that *R*(*x*,*t*) is overestimated by 15–20% (Fig. 2b), as the model does not includes the viscosity of the capillary bridge and a purely inertial event is considered. To include the viscous dissipation in the cavity formation we start the derivation from the cavity expansion formed after a single droplet impact on a pool. By using potential flow theory and considering shear stresses in a thin layer covering the interface of the cavity, the cavity evolution can be expressed by the following system of equations,^53^5
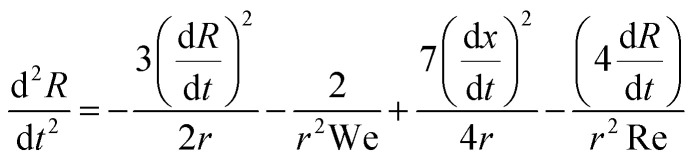
6
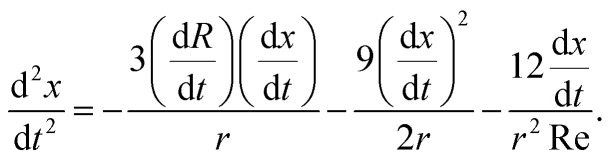


Similarly to Speirs *et al.* 2018,^52^ we use the resemblance of a cavity generated by a train of droplets and liquid jet. We use the solution of the cavity expansion of a single droplet given by eqn (5) and (6), and at a given time we superimpose the solution of the expansion of the next impacting droplet. This allows us to predict the cavity evolution produced by a jet in a viscous capillary bridge. To generate a comparable cavity to that of a jet, the train of droplets needs to have a similar volumetric flux as a jet. Therefore, we equate the volume of a droplet 
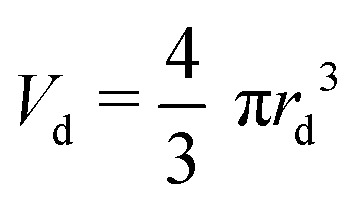
 to that of a cylinder that encapsulates a train of droplets, *V*_cyl_ = π*r*_cyl_^2^*h*_cyl_,7
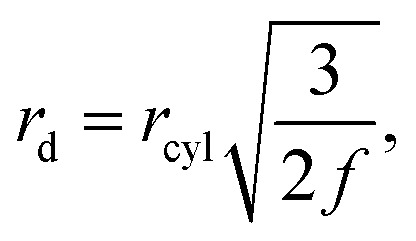
where *h*_cyl_ = 2*r*_d_/*f* is the droplets centre-to-centre distance, *f* is the frequency of the train of droplets and *r*_cyl_ is the cylinder radius. Furthermore, previous work has shown that the cavity velocity is dependent on the frequency of the droplet train,^50^8
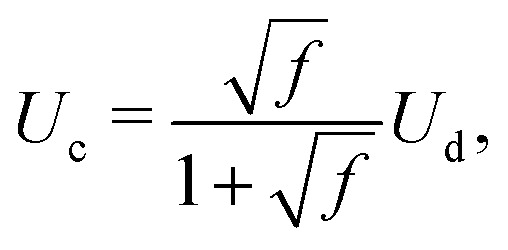
where *U*_d_ is the droplet velocity. Therefore, for a train of droplets to have a similar cavity velocity than for a jet with velocity *U*_0_ we need,9
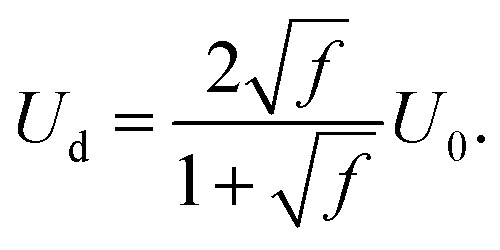


**Fig. 3:**
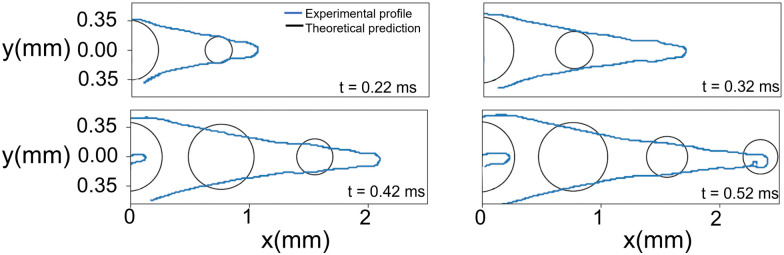
Comparison between the experimental cavity profile (blue line) and the theoretical profile for the droplet train (black circles). The experimental conditions are the same as for the impact on glycerol 78% in Fig. 2. The ‘finger’ shown at times 0.42 and 0.52 ms is the result of subsequent droplets entering the field of view.

We take the corrections of eqn (7) and (9) for a train of droplets with a frequency *f* = 1/2, and overlay it to the experimental profiles of glycerol 78% and show the results in Fig. 3. As observed in Fig. 3 the agreement of the expansion at times *t* = 0.22 ms and *t* = 0.32 ms is excellent. In contrast, the expansion radius *r*(*x*) is slightly over-predicted for times *t* = 0.42 ms and *t* = 0.52 ms. However, overall the model of the train of droplets provides a better estimate of *r*(*x*) than the use of the two-dimensional Rayleigh equation, where the viscous dissipation is neglected.

For the PEO 600k 1 wt% capillary bridge, the theoretical cavity profile fits well with the prediction in eqn (4) close to the tip of the cavity (Fig. 2). However, we observe bulging close to the impact point which cannot be easily explained. We hypothesise that, given the shear thinning nature of PEO (see the ESI,† Fig. S1) close to the impact point the effective viscosity is lower than that after the jet decelerates during the full experiment. The shear rate during the impact can be calculated as *γ̇* ∼ *U*_0_/*D*_0_,^54^ thus, for our experiments we obtain *γ̇* ∼ 10^5^ s^−1^. Consequently, at the entry point the radial expansion of the PEO 1 M and 600k 1 wt% cavities is more efficient than at the end of the process. Furthermore, elastic stresses have been found to enhance the expansion ratio for viscoelastic liquids compared to Newtonian liquids of the same viscosity.^55–57^ This could further explain the bulging after the impact on PEO solutions and that the theoretical model underpredicts the cavity radius near the impact point. The cavity also shows a smoother profile compared to that of water. The latter can be expected as PEO 600k 1 wt% has a viscosity ≈20 times higher than water, damping the capillary waves.

In addition to studying the cavity geometry during expansion, we tested the model for the critical velocity of a jet traversing a liquid droplet that we developed previously.^46^ Assuming that the surface tension dominates during the cavity collapse and using the shape of the cavity described by eqn (4) we can predict the time of the collapse of the cavity,^46^10
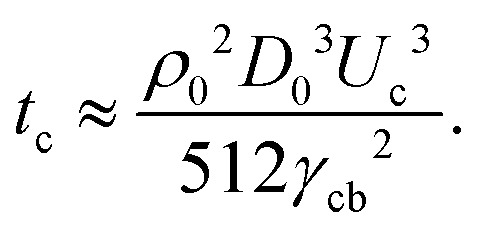


Then if the diameter of the capillary bridge *D*_cb_ is larger than *H*_max_ = (*U*_0_/2)*t*_c_ the jet would traverse the droplet. Rearranging so that we have a critical Weber number for the traversing, we obtain,11
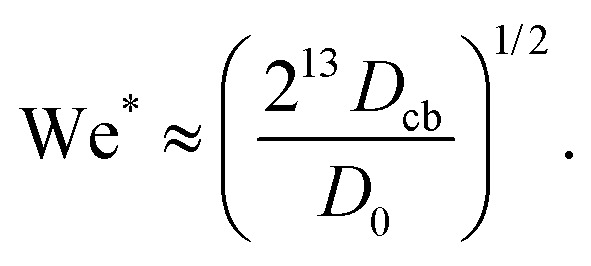


To compare this result with the experimental data, we use the ratio between the critical traversing Weber number We* and the experimentally measured one We, *i.e.*,12
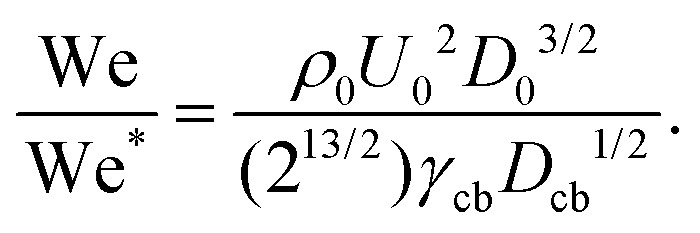


**Fig. 4:**
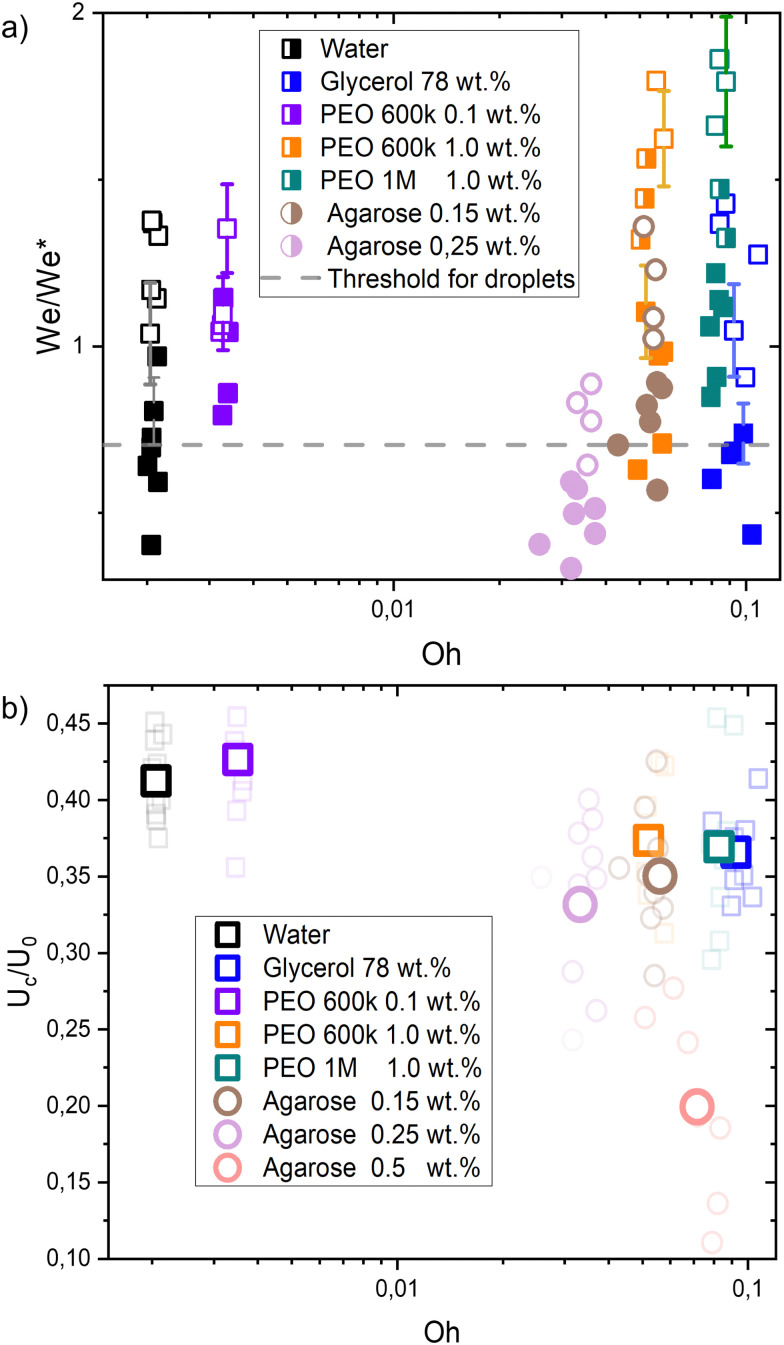
(a) Phase diagram comparing the critical Weber number for traversing obtained experimentally (We) and from eqn (11) (We*). Each liquid and agarose gel are represented by their respective Oh number. Open symbols show cases where the jet traverses the droplet, while solid ones stand for the embedding case. The dashed line is We/We* = 0.7 the threshold found for droplets in ref. 46. The model is in good agreement with the experimental data for liquids and agarose 0.15 wt%. In contrast, the threshold is underestimated for the agarose 0.25% wt and 0.5% wt (not shown due to scale). Uncertainty was calculated for all the experimental data and example error bars are shown at selected points. (b) Ratio of the cavity velocity *U*_c_ and the impact velocity *U*_0_, for the liquids and agarose gels studied in this experiment. For each liquid, bold coloured symbols represent average values of light symbols. Even for liquids with Oh < 0.01, *U*_c_/*U*_0_ is lower than the predicted 0.5, and we attribute this to the wall dissipative effects.

Fig. 4a shows a comparison between the threshold predicted by eqn (12) and the threshold found in our experiments. The Ohnesorge number (
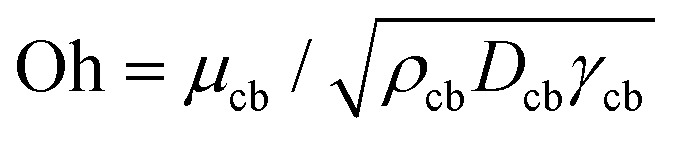
, with subscript cb indicating the capillary bridge properties) represents the ratio between viscous to inertial and capillary forces. The threshold for traversing the Newtonian capillary bridges is within 10% of the model prediction. In contrast, for PEO 600k and 1 M 1.0 wt% the threshold deviates in ≈50%. As previously shown,^46^ the penetration threshold is influenced by the relaxation time considering De = *λ*/*τ*_c_, where *τ*_c_ = (*ρ*_cb_*D*_cb_)/*γ*_cb_.

In Fig. 4 the dashed line shows the experimental threshold between embedding and traversing for the impact of a microfluidic jet on pendant droplets.^46^ We find that the threshold for traversing Newtonian capillary bridges is higher than for pendant droplets. This is a consequence of assuming that the cavity advances at a constant velocity *U*_c_ = 1/2*U*_0_. However, as shown in Fig. 4b, *U*_c_ < 1/2*U*_0_ for all the capillary bridges. We attribute this decrease in *U*_c_ to the confinement, provided by the glass holding the capillary bridge. Indeed, in previous experiments the influence of confinement has been found to reduce the cavity radial expansion and increase the energy dissipation.^26,27,41,58^ Moreover, in Fig. 4b we show that the velocity of the expanding cavity depends on both the viscosity and density ratios of the target and projectile. By considering viscous dissipation (*μU*_0_^2^/*D*_0_^2^), and balancing the initial kinetic energy of the jet with the displaced liquid on the pool and energy of the jet at a time *t* after impact, then *U*_c_ can be expressed as,^59^13
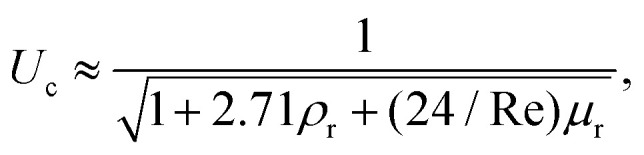
where *ρ*_r_ and *μ*_r_ are the density and the viscosity ratios between the microfluidic jet and the capillary bridge.

For agarose 0.15 wt%, the prediction of the traversing threshold is within the same range of values as for the liquids. For agarose 0.25 wt%, the threshold is of the same order of magnitude as for liquids, but differs by ≈30%. Furthermore, the threshold found for agarose 0.5 wt% is one order of magnitude lower than the predicted value (not shown in the plot). The average measured *U*_c_/*U*_0_ for agarose 0.5 wt%, is five times lower than the one used to predict the traversing, however this difference is insufficient to explain the discrepancy.

That the predicted transition occurs at a lower We/We* for increasing agarose concentration seems contradictory as for increasing the elastic modulus there is an increase in viscous dissipation.^48^ This underestimation of the traversing threshold could be attributed to an overestimation of the surface tension for agarose gels with *G* ≳ 500 Pa. The linear relationship used for calculating the surface tension and viscosity were obtained for agarose gels with concentrations lower than 0.25 wt% and with *G* < 200 Pa.^48^ Therefore we see a good agreement with the agarose 0.15 wt% (which *G* = 176), but possibly for gels with *G* > 500 Pa the linear relation is no longer valid. We expect that this result translates to skin or other complex materials, where surface tension and viscosity are difficult to assess and define.

### 3.2. Cavity collapse

#### Closure regimes

**Fig. 5:**
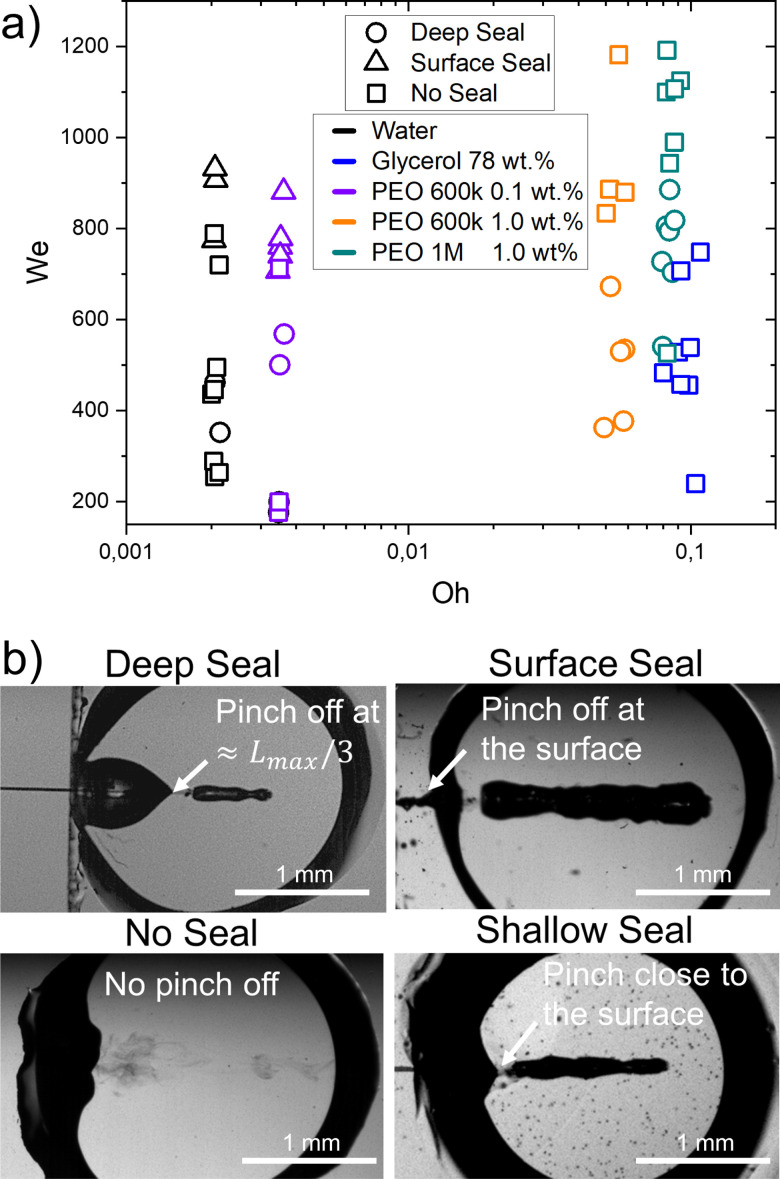
(a) Different types of seals as a function of We and Oh. The material properties influence the type of collapse observed at the same Weber numbers. Experimental snapshots of the different type of closure are shown on the right. Movies 1–3 in the ESI† show the ‘no seal’ regime. Movies 4 and 5 represent ‘deep seal’ and ‘surface seal’ regimes respectively. (b) Experimental images showing the four seal regimes.

We classify the closure type of the cavities in four categories: ‘no seal’, ‘surface seal’, ‘shallow seal’ and ‘deep seal’, as shown in Fig. 5b.^22,24^ The type of seal depends on the capillary bridge properties as well as on the Weber number.

In the ‘no seal’ regime, the cavity stops at *L*_max_ or at *D*_cb_ in the case of traversing and retracts until the capillary bridge returns to a circular shape. In addition, we define the ‘deep seal’ regime as the phenomena where the cavity collapses at a pinch point larger than *L*_max_/3. Furthermore, within the ‘shallow seal’ regime, the cavity collapses close to the jet entry point. Moreover, the ‘surface seal’ regime refers to the closure of the surface when the cavity is still expanding, and an overarching dome that closes at a location *x* < *x*_0_, where *x*_0_ is the impact point.

During the ‘no seal’ regime, in contrast to the impact of droplets on liquid pools or a jet on a pendant droplet, no Worthington jets are observed. We attribute this to the reflection of the surface waves on the glass walls as it was found that capillary waves control the cavity pinch-off and Worthington jet properties.^12,17,60^ Furthermore, at the base the cavity can expand until contacting the walls. This causes the liquid to wet an area larger than that of the initial capillary bridge. Glass is wettable and therefore the contact line gets pinned into the walls that contain the liquid capillary bridge. Thus, the cavity retraction suffers from energy loses due to viscous and contact line dissipation and a Worthington jet is no longer energetically favourable. A more detailed discussion on the wettability effects of the walls is presented in Section 3.3. Furthermore, for this regime to occur the aspect ratio of the cavity at the onset of retraction, the length of the jet and the We number are crucial parameters to consider.^12^ In this type of retraction no bubbles are trapped inside the capillary bridge. In the ‘deep seal’ regime, after the cavity pinches-off, the cavity may further pinch-off at multiple locations. Therefore, multiple bubbles and antibubbles are trapped inside the capillary bridge, where antibbubles are defined as droplets with an air shell.^61^ We observed this type of sealing regime for an increasing Weber number with an increasing PEO molecular weight and concentration. For PEO 600k 0.1 wt% deep seal is observed for We ≈ 500–600, for PEO 600k 1.0 wt% We ≈ 400–700 and for PEO 1 M 1.0 wt% We ≈ 500–900. After increasing the PEO molecular weight and concentration the PEO solutions are more viscous and have a larger relaxation time. Consequently, the cavities are smaller at the same We for increasing molecular weight and concentration. Lastly, we observe that viscoelasticity favours the ‘deep seal’ regime. Indeed, glycerol 78% has a similar Oh as compared to PEO 1 M 1.0 wt%, but this type of seal is not observed. Within the ‘shallow seal’ regime, the cavity collapses close to the jet entry point. This type of seal was observed for all the agarose capillary bridges at all the explored conditions, while it was absent in Newtonian and viscoelastic capillary bridges. This regime is characterised by the entrapment of several bubbles. The ‘surface seal’ regime refers to the closure of the surface when the cavity is still expanding, and an overarching dome that closes at a location *x* < 0. The latter is observed for We ≲ 700 and low viscosity liquids (Oh ≲ 0.004). This regime has been studied in detail in ref. 13 and 22.

#### Cavity profiles

**Fig. 6:**
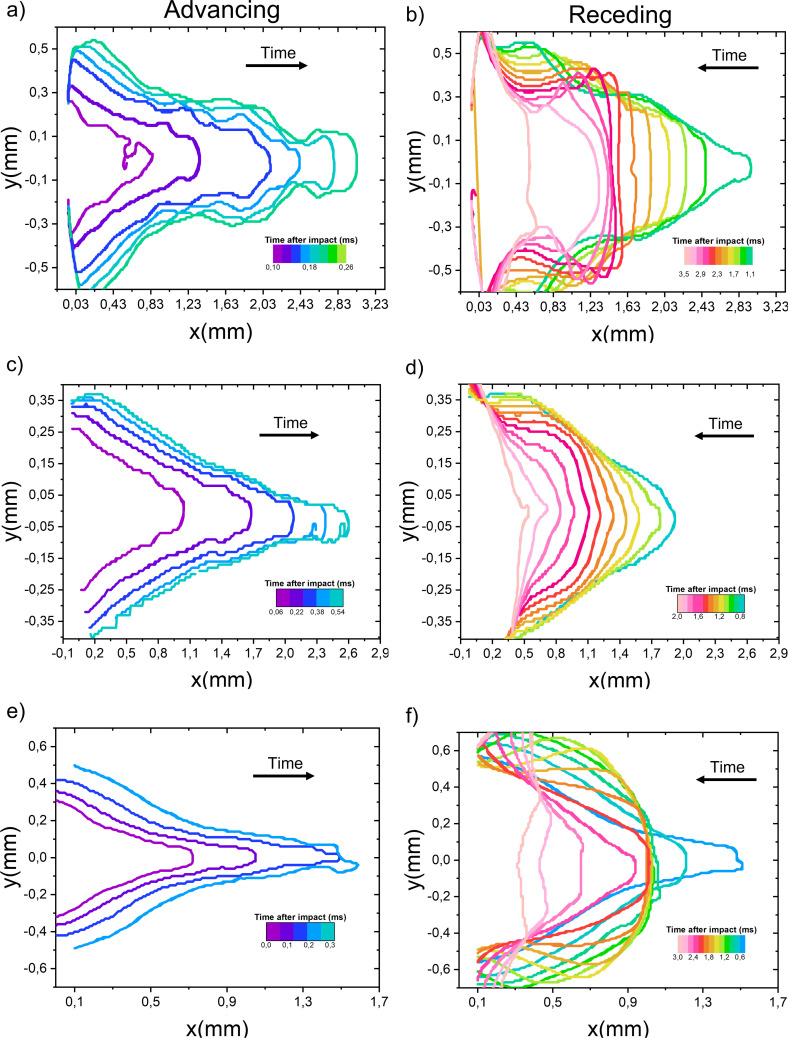
Cavity profiles after the impact of a microfluidic chip on a capillary bridge. (a and b) Water capillary bridge; we observe capillary waves travelling across the cavity throughout the process. (c and d) Glycerol 78 wt%. capillary bridge; the capillary waves are damped due to viscous dissipation. (e and f) PEO 600k 1 wt% capillary bridge; while the cavity is receding the cavity front stops from *t* ≈ 1.8 ms to *t* ≈ 2.4 ms and the tip of the cavity stays roughly at the same position. The experimental conditions are the same as in Fig. 2.

We study now how the area of the cavity during the collapse stage is affected by the liquid properties and the cavity profiles at different times. In Fig. 6 we show the cavity profiles of different liquids during advancing and receding for We ≈ 490. For the water capillary bridge, propagating surface waves can be observed for both the advancing and receding of the cavity (Fig. 6a and b). In contrast, for the aqueous glycerol mixture capillary bridge, the propagating waves are damped by the viscosity of the liquid.^62^ Furthermore, for the PEO600k wt1% capillary bridge we observe that the front of the cavity stops at an instant comparable to the polymer relaxation time (*t* ≈ *λ* ≈ 1.5 ms). This retardation in the collapse has been observed for voids generated in a viscoelastic fluid and it was attributed to the liquid elasticity.^63^

Fig. 6, also shows that the *R*(*x*,*t*) is the smallest for the glycerol 78% which can be explained from eqn (5) and (6) as discussed in Section 3.1. Since the data in Fig. 6, is taken at a constant Weber number, we conclude that the maximum area of the cavity *A*_max_ depends on the liquid viscosity. As noted in Section 3.1, viscosity opposes the radial expansion of the cavity, limiting *A*_max_. Furthermore, the PEO 600k 1 wt% has the smallest *L*_max_. This is in line with previous work where it was found that a viscoelastic liquid makes a bullet decelerate faster than purely viscous or shear-thickening liquids with the same zero shear viscosity.^23^

**Fig. 7:**
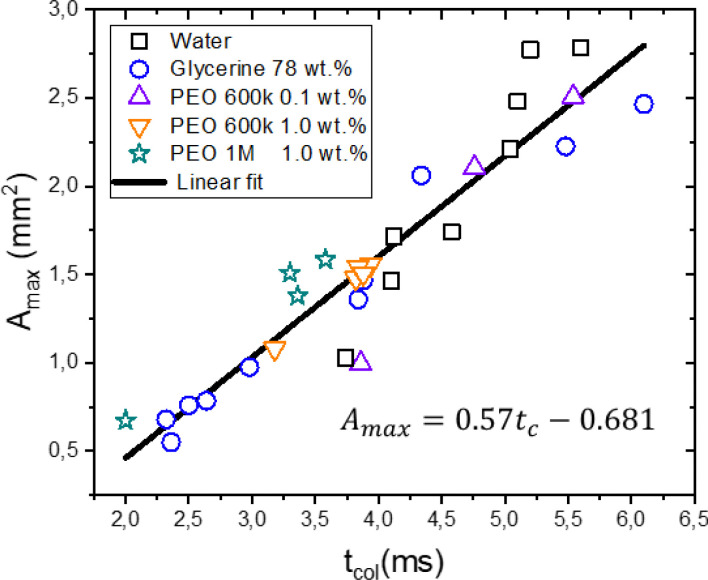
Maximum cavity area *A*_max_ as a function of the collapse time *t*_col_. There is a linear correspondence between *t*_col_ and *A*_max_ for all the liquids, indicating that the cavity retraction and collapse is a surface tension driven process.

Fig. 7 shows the maximum cavity area *A*_max_ as a function of the collapse time *t*_col_ for all the liquids and cavities collapsing within the no seal regime. From this figure we observe that *t*_col_ follows a linear relationship with *A*_max_. Given that all liquids have a similar surface tension, the latter indicates that cavity retraction and collapse is driven by surface tension. At first glance it might be surprising that the time of collapse is independent of viscosity. However, the damping effect of viscosity on the capillary waves shelters the collapse from other perturbing ripples.^64–66^ This allows for a smoother collapse for glycerol 78 wt% than for water, resulting in a similar collapse time for a given area. A similar trend has been found for the retraction of cavities generated in droplets^46^ and liquid pools.^17^

#### Entrained bubbles

**Fig. 8:**
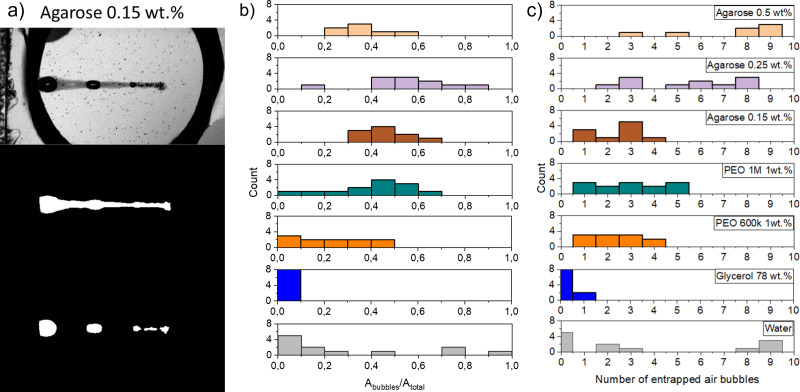
Characterisation of bubbles entrained after injection. Total area of entrapped bubbles (*A*_bubbles_), total injected area (*A*_total_) and number of entrapped bubbles for each capillary bridge. (a) Image analysis used to extract *A*_total_ and *A*_bubbles_. Top panel: raw image; middle panel: total injected area (*A*_total_); bottom panel: area of bubbles *A*_bubbles_. (b) Histogram showing the ratio of the bubble area to total injected area for the liquid and agarose capillary bridges at different impacting conditions. The ratio depends on the closure characteristics. For the ‘no seal’ events no trapped bubbles are observed and in consequence all the injected area is conformed by the liquid jet, *e.g.*, for glyecerine 78 wt%. In contrast, for surface seal events most of the area of the cavity is entrapped as a bubble, thus, the ratio of *A*_bubbles_/*A*_total_ is close to 1. (c) Histogram showing the frequency of the number of entrapped droplets for each liquid at different impacting conditions. The number of trapped air bubbles depends on closure characteristics. Several (≈4–10) trapped bubbles are the result of the cavity pinching off in different locations. While one or two entrapped bubbles are an indication of the cavity pinching off before the whole volume of the jet is injected into the capillary bridge. In the case of an ideal injection, no bubbles would be trapped and *A*_bubbles_/*A*_total_ = 0, *i.e.*, it would look similar to the injections at the glycerol capillary bridge.

We investigate now the number and characteristics of entrained bubbles after jet impact on the different capillary bridges. We compare the area of liquid that remains inside the capillary bridge (*A*_total_) with the area of the entrained bubbles *A*_bubbles_. An example of the image analysis to obtain *A*_total_ and *A*_bubbles_ is given in Fig. 8a. *A*_total_ is simple to calculate because it is darker than the background, and bubbles show as darker spots within *A*_total_. Since the minimum bubble diameter our optical system can detect is 100 μm, clusters of bubbles with a smaller diameter than our resolution are taken as a single bubble with the combined area of the cluster. The first panel of the figure shows the last frame of the impact of a jet on an agarose 0.15 wt% capillary bridge. The second panel shows the binarised image with *A*_total_, that counts both the area of the injected liquid and *A*_bubbles_. The last panel shows the binary image showing *A*_bubbles_ only. These parameters give information about the efficiency of the injection, for example in needle-free applications where air entrainment is undesirable.^67,68^

In Fig. 8b we show the ratio between the total area and the area of the entrapped bubbles *A*_bubbles_/*A*_total_, for the different capillary bridges. A ratio of 0 indicates no bubble entrapment, while a ratio of 1 implies that just bubbles are entrapped and no liquid is injected. The total number of entrapped bubbles depends on the capillary bridge properties, such as viscosity and the closure regime, which in turn is related to the Weber number. Fig. 8c shows the number of entrained bubbles for all the experiments. The difference between the agarose samples at 0.25% and 0.5% are statistically significant from the rest of the materials (*p*-value = 1.04057 × 10^−4^), but not between themselves (*p*-value = 0.10565). Furthermore, the number of bubbles entrained in Agarose 0.15 wt% and all the liquids, excepting glycerol 78 wt%, are statistically similar (*p*-value = 0.61004043).

For water capillary bridges, *A*_bubbles_/*A*_total_ ranges from 0 to 1. This is the result of the different cavity collapse regimes in water depending on the Weber number (Fig. 5a). For example in a ‘surface seal’ the injection results in a bubble area comparable to the maximum cavity area *A*_max_. In contrast, in the ‘no seal’ regime most of the injected volume comes from the jet and no bubbles are entrapped.

In the case of glycerol capillary bridges, the entrapped air at the end of the injection is negligible, as in all the experiments the cavity collapse was in the ‘no seal’ regime (Fig. 5a). Since all the liquid of the jet is injected into the capillary bridge, this is the ideal situation for needle-free injection applications.

PEO solutions present the cavity collapse regimes of ‘deep seal’ and ‘no seal’. In the ‘deep seal’ regime the cavity collapses after some liquid is already stationary inside the capillary bridge. Consequently, *A*_bubbles_/*A*_total_ did not exceed 0.6. Interestingly, the distribution of *A*_bubbles_/*A*_total_ and number of entrained bubbles of PEO 1 M 1 wt% is similar to the distribution in agarose 0.15 wt%. We attribute the similarity to the resemblance in cavity shape and sizes in both of the materials.

For agarose capillary bridges *A*_bubbles_/*A*_total_ is always close to ≈0.5. This is a consequence of only the ‘shallow seal’ regime being observed for these gels. In addition, the size of the trapped bubbles in agarose is in general smaller than on liquids. This is explained by the cavity collapse at multiple points before the end of the injection. Furthermore, the area of the cavity is smaller for agarose capillary bridges as compared to liquid ones.

The studies on trapped bubbles are relevant to needle-free applications, especially on agarose gels as they are widely used as skin surrogates.^39,69^ Therefore, the consideration of trapped bubbles and how to minimise their occurrence and volume need to be addressed, when injecting onto real skin. We can conclude that preventing the cavity collapse at the surface of the injection is crucial to avoid entrapping bubbles larger in volume than the injected liquid. However, as skin resembles more agarose than water, and we did not observe surface seal for agarose, we deem the ‘surface seal’ regime unlikely for skin. Furthermore, by promoting the ‘no seal’ regime, all the liquid of the jet would be injected without air entrapment.

### 3.3. Surface wettability effects

**Fig. 9:**
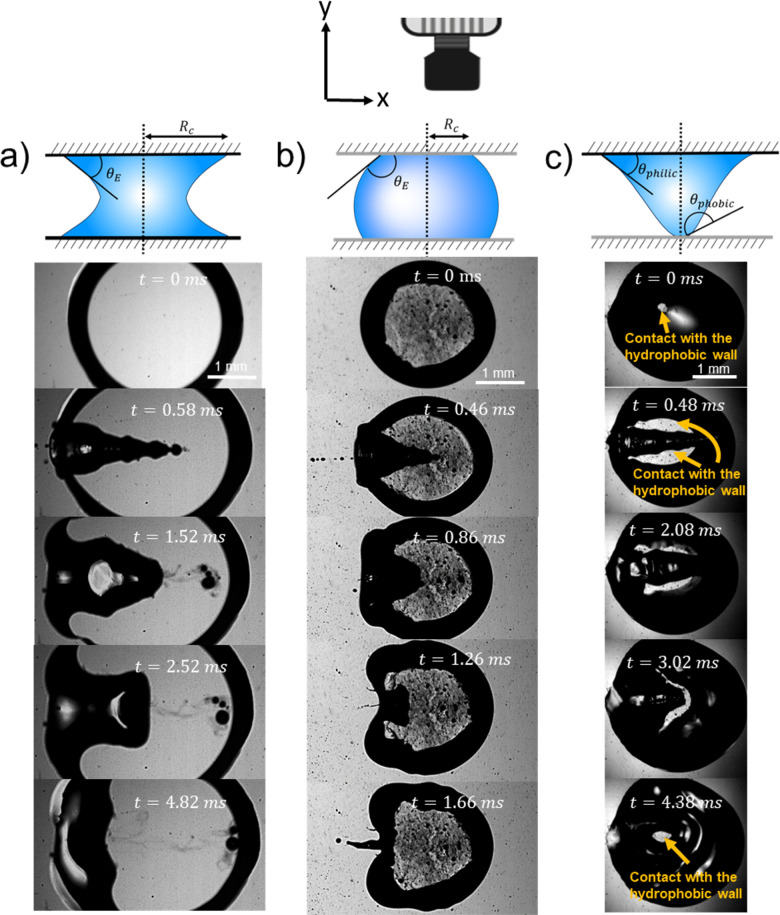
Schematics of the capillary bridge geometry and snapshot sequences of the retraction of the cavity generated after the impact of a jet on a capillary bridge. In (b) a schematic of the camera and the reference system is shown to illustrate that we take the experimental images from the top. (a) Capillary bridge between hydrophilic surface (We = 352; Movie 6 in the ESI†). In this case the capillary bridge is concave. (b) Capillary bridge between hydrophobic surfaces (We = 325; Movie 7 in the ESI†). In this case the interface of the liquid bridge is convex. (c) Capillary bridge with a hydrophilic surface on the top and a hydrophobic surface on the bottom: hydrophilic–hydrophobic (We = 320; Movie 8 in the ESI†). Here, the capillary bridge changes from curvature and adopts a *conical* shape. After the impact, for the hydrophobic surfaces, the contact line moves and a Worthington jet is observed. In contrast, for the hydrophilic surfaces, as well as for the hydrophilic–hydrophobic surfaces the contact line remains pinned and no Worthington jet is observed.

**Fig. 10:**
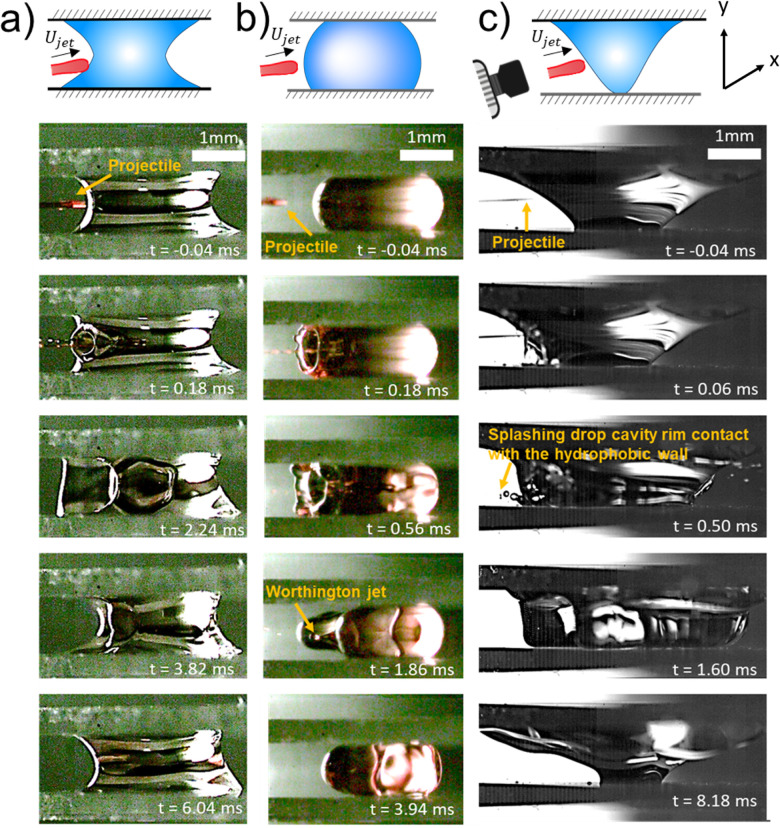
Snapshot sequences of the front view of the impact of a jet onto a capillary bridge. In (c) a schematic with reference system of the camera is placed to show the angle from which the experimental images were obtained. (a) Capillary bridge between hydrophilic surfaces (We = 468). At *t* = 0.18 a cavity is created with its respective rim. While the cavity expands, the rim touches the walls and remains pinned (Movie 9 in the ESI†). (b) Capillary bridge between hydrophobic surfaces (We = 408). In this case, when the rim touches the walls, it bounces back. Due to this bouncing the contact line moves. At time *t* = 1.28 ms droplets coming from the Worthington jet can be observed (Movie 10 in the ESI†). (c) Capillary bridge with a hydrophilic surface on the top and a hydrophobic on the bottom: hydrophilic–hydrophobic (We = 1563). Here, when the rim grows and contacts the walls, the liquid pins in the hydrophilic wall, while it splashes in the hydrophobic wall. At time *t* = 1.60 ms most of the liquid detaches from the hydrophobic wall, and at time *t* = 8.18 ms, the liquid bridge is almost at the equilibrium position (Movie 11 in the ESI†).

In this section we change the wettability of the walls where the capillary bridge is contained and assess its effect on the impact process. We applied Glaco coating on the glass walls (originally hydrophilic) to render them hydrophobic and we used water for the capillary bridges. Fig. 9 and 10 present snapshots of the experiments in the perpendicular view and at an angle. In Fig. 9 we show the retraction dynamics of the cavity formed after a jet impact on a capillary bridge that is between either hydrophobic walls (Fig. 9a), hydrophilic walls (Fig. 9b) or a mix hydrophilic on the top and hydrophobic on the bottom (hydrophilic–hydrophobic, Fig. 9c). The cavity retraction ends with a Worthington jet on the hydrophobic walls. In contrast, no Worthington jet was observed during the retraction on hydrophilic and hydrophilic–hydrophobic walls. In these cases we define a Worthington jet as such formed after the cavity collapse that results in at least one ejected droplet. Worthington jets were observed for ≈30% of the experiments for hydrophobic walls. In contrast, the experiments for hydrophilic walls led to Worthington jets in ≈3% of observations, and in none of the experiments between hydrophobic–hydrophilic walls. In the latter, the wetted area between the top and the bottom cavity is different. When the cavity expands, part of the liquid of the capillary bridge impacts the hydrophobic wall, and bounces towards the hydrophilic wall during line recession (see Movie 11). Thus, the cavity collapses asymmetrically, explaining the absence of the Worthington jet on the hydrophobic–hydrophilic walls configuration. Additionally, we observed that the change of wettability is negligible during cavity expansion, as expected from an inertia dominated process (see Section 3.1). The only difference being the fate of the rim of the cavity when it touches the wall, where it can stay pinned or bounce (see Fig. 9 and Movies S9–S11, ESI†).

To understand the observed difference in the contact line dynamics between hydrophilic and hydrophobic surfaces, first, we study the hydrophilic–hydrophobic capillary bridge. In this configuration, before impact, the liquid is touching only a point on the hydrophobic surface, while wetting an area of 6.3 mm^2^ on the hydrophilic wall (see Fig. 9c at *t* = 0 ms and the first panel of Fig. 10c). During cavity expansion, the liquid of the capillary bridge moves from the impact point towards the hydrophobic wall and wets it (see Fig. 9c at *t* = 0.48 ms). If the impact force is enough the rim will splash after contacting the hydrophobic wall as seen in Fig. 9 and Video S11 (ESI†). When the cavity recedes and the capillary bridge reaches equilibrium the liquid dewetts the wall and its state is similar than before the impact. In contrast, the contact line stays pinned in the hydrophilic wall for the whole process.

Fig. 9 shows the geometry of the capillary bridge for hydrophobic, hydrophilic and hydrophilic–hydrophobic walls. We note that the liquid bridge is concave for hydrophilic walls and convex for hydrophobic walls. For the hydrophilic–hydrophobic walls the capillary bridge changes from curvature near the walls and adopts a conical shape. These curvature changes are the result from the equilibrium contact angle of the surfaces and surface energy minimisation. The difference in curvature introduces variations in the Laplace pressure Δ*P* between the different capillary bridges. The Laplace pressure of a capillary bridge of height *H* with a contact angle *θ*_E_ is^70^,14
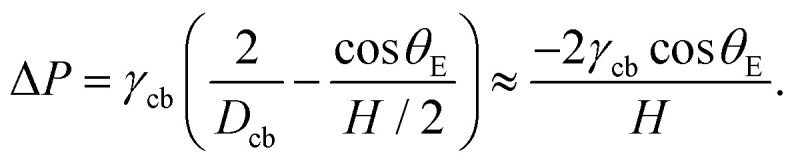


From eqn (14), we observe that the Laplace pressure is positive for a convex bridge and negative for a concave bridge as for cos(*θ*_E_) > 0 for *θ*_E_ < π/2 and cos(*θ*_E_) < 0 for *θ*_E_ > π/2.^70^

The vertical adhesive force *F*_a_ of the liquid acting on the walls *F*_a_ is given by the Laplace pressure and the axial component of the surface tension acting along the contact line.^71,72^ The contact line force *F*_cl_ is given by the sine of *θ*_E_, the perimeter of the contact area 2π*R*_c_ and the surface tension of the capillary bridge *γ*_cb_. Thus, the vertical adhesive force can be written as^73^,

15 *F*_a_ = 2π*γ*_cb_*R*_c_sin*θ*_E_ − π*R*_c_^2^Δ*P*

where *R*_c_ is the contact radius of the capillary bridge. The contact radius is also influenced by the surface wettability and the same liquid volume *R*_c_ is larger for a hydrophilic surface than for a hydrophobic one. A typical contact radius for a 2 μL droplet on glass surfaces is ≈2.5 mm, while for a water droplet on the Glaco coated surfaces is ≈1.9 mm. For positive *F*_a_, the liquid is attracted by the walls, while negative values of *F*_a_ suggest repulsion from the walls.^73^ In our experiments glass has a contact angle of *θ*_E_ = 23 degrees with water, and the Glaco coated glass has a contact angle of *θ*_E_ = 160 degrees with water. Therefore, for our experiments a typical adhesion force on a capillary bridge between glass walls is ≈3 mN and for Glaco sprayed glass is ≈−1.2 mN. During a fluid–fluid displacement system there is significant energy dissipation from the contact line.^58^ In particular, when a system is confined, the ratio of the interfacial area to bulk volume increases, rendering the contact line dissipation more prominent. Contact angle hysteresis, defined as the difference between the advancing and receding contact angles (*θ*_a_ and *θ*_r_ respectively), is responsible for the adhesion of drops to inclined surfaces and determines the contact line dissipation *Φ*, which can be written as,^74,75^

16 *Φ*_cl_ = 2π*R*_c_*γ*_cb_(cos *θ*_a_ − cos *θ*_r_)

Hydrophilic surfaces generally have a larger contact angle hysteresis than hydrophobic surfaces.^76^ More specifically, as for Glaco sprayed surfaces water rests on a thin air layer, liquids are very mobile, and there is an almost negligible contact angle hysteresis.^76^ In contrast, surface heterogeneity causes hysteresis in water, we measured hysteresis to be ≈18 degrees. From equation 16 we determine the contact line dissipation in the glass surfaces to be ≈0.08 mJ, which is comparable to the kinetic energy of the jet *E*_kjet_ ≈ (π/8)*ρ*_0_*U*_0_^2^*D*_0_^2^*L*_0_ ≈ 0.12 mJ, where *L*_0_ is the length of the jet. Consequently, we expect that the jet does not have enough energy to make the contact line dewett the walls.

In summary, the Worthington jet is not energetically favourable for the capillary bridge confined between hydrophilic surfaces. The contact line dissipation and adhesion forces in this configuration are comparable to *E*_kjet_. In contrast, for hydrophobic walls, the adhesion force and contact line dissipation are negligible, allowing contact line movement, thus a Worthington jet becomes energetically favourable.

## 4. Conclusions

This work presented experimental results on the ballistics of a microfluidic jet impacting on liquid and agarose capillary bridges. By using high speed imaging and image analysis we extracted the cavity profiles, which allowed us to understand in detail the impact dynamics for each material.

We modelled the cavity expansion and shape based on the comparison between the Young-Laplace and the dynamic pressures of the cavity made by the penetrating jet. We then compared the model of the cavity shapes (summarised by eqn (4)) with experiments, finding good agreement between the experiments and model for the cavity shape and evolution on the water capillary bridge. However, for the impact on the glycerol capillary bridge we observed that the radial cavity expansion is overestimated by ~15–20%. We attribute this mismatch to viscous losses, which are neglected in eqn (4). By using eqn (5) and (6) and the simile between a jet and a droplet train, we arrived to an expression that predicts the cavity profile that depends on the Reynolds number.

Furthermore, we found that the Weber number threshold for traversing a capillary bridge is larger than for a pendant droplet. We attributed this to the confinement and dissipation of energy along the walls. Eqn (12) predicts the threshold with 10% accuracy for Newtonian liquids and the agarose gel 0.15 wt%. In contrast, for agarose gel 0.5 wt%, the predicted threshold is smaller by one order of magnitude. We attribute this deviation to an overestimation of the surface tension predicted by eqn (1). This indicates that as the shear modulus of the gels is increased, they can no longer be treated as liquids. Hence, for accurately modelling the interaction between the jet and soft solids with a storage modulus larger than 536 Pa, more complex models need to be implemented. For example, measuring the shear forces and stress during the jet penetration into the agarose gels.

Additionally, we studied the cavity collapse, the number of trapped bubbles and the ratio between the area of the bubbles and the injected liquid. For the range of Weber numbers studied here, the water cavities presented a varied type of collapse. Consequently, the number of trapped bubbles varied from 0 to 10 and *A*_bubbles_/*A*_total_ was in the range from 0 to 1. The aqueous glycerol solution had a single collapse mode and no bubbles were trapped. Finally, for the agarose gels, the cavity was observed to collapse in multiple points trapping several bubbles that amounted to an area approximately half of the injected area. Accordingly, for needle-free injections, the ideal scenario is the ‘no seal’ regime. In contrast, the surface seal regime should be avoided. However, as skin is viscoelastic and has a similar storage modulus to agarose, we do not expect this scenario to happen in the range of velocities leading to injection.

Finally, we assessed how the wettability of the walls confining the capillary bridge influenced the cavity collapse. We argue that the Worthington jet is suppressed for the hydrophilic walls due to a larger adhesion force (eqn (15)) and the contact line dissipation of energy (eqn (16)), as compared to the hydrophobic walls.

Our results show that for inertial injection processes, agarose gels with modulus around 176 Pa can be treated as liquids, thus connecting physical principles between liquids and soft solids. However, further research needs to be carried out to bridge the gap between liquids and soft solids with a storage modulus larger than 536 Pa. Furthermore our study provides insight on the amount of liquid and trapped bubbles generated after the impact of a microfluidic jet onto a confined target. Better control and understanding of how the cavity collapses presents an important advance towards the control of needle-free injections.

## Author contributions

M. A. Q. S. and D. F. R. conceived the experiments, designed and built the experimental setup, analysed the results and wrote the manuscript. M. A. Q. S. performed the experiments.

## Conflicts of interest

There are no conflicts to declare.

## Supplementary Material

SM-019-D2SM01285E-s001

SM-019-D2SM01285E-s002

SM-019-D2SM01285E-s003

SM-019-D2SM01285E-s004

SM-019-D2SM01285E-s005

SM-019-D2SM01285E-s006

SM-019-D2SM01285E-s007

SM-019-D2SM01285E-s008

SM-019-D2SM01285E-s009

SM-019-D2SM01285E-s010

SM-019-D2SM01285E-s011

SM-019-D2SM01285E-s012
