# Supplementary material for: Cavity dynamics after the injection of a microfluidic jet onto capillary bridges
Source: Soft Matter. 2022 Dec 1;19(2):245–57. doi: 10.1039/d2sm01285e (PMC9811641; doi:10.1039/d2sm01285e)
Supplement: SM-019-D2SM01285E-s004 [file SM-019-D2SM01285E-s004.pdf]

Electronic Supplementary Material (ESI) for Soft Matter.
This journal is © The Royal Society of Chemistry 2022

Electronic Supplementary Information for Soft Matter manuscript: Cavity dynamics after the injection of a microfluidic jet onto capillary bridges

Miguel A. Quetzeri-Santiago[a] and David Fernandez Rivas

[a] Mesoscale Chemical Systems Group, MESA+ Institute and Faculty of Science and Technology, University of Twente, P.O. Box 217, 7500AE Enschede, The Netherlands

Figures in Supporting Information

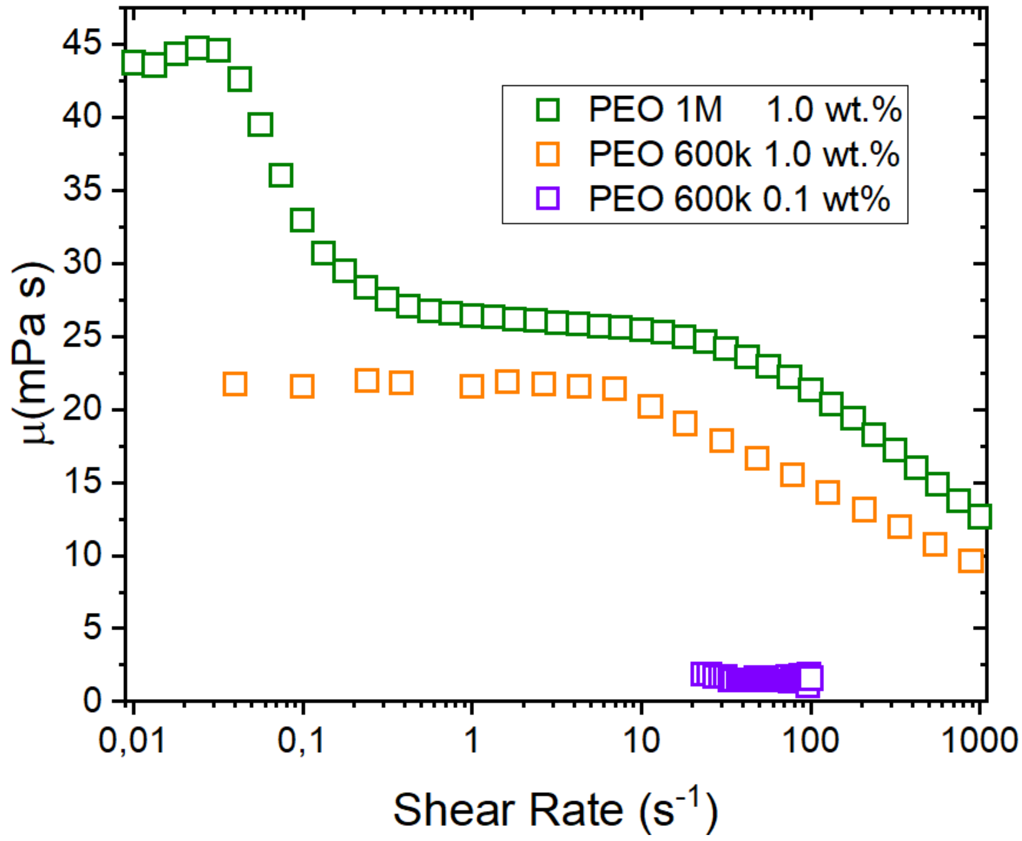

Fig S1. Rheology of aqueous PEO viscoelastic solutions of different molecular weights PEO 1M at 1 wt.% and PEO 600k at 1 wt.% and 0.01 wt.%. The measurements show a constant zero shear viscosity for shear rates 0.01-0.03 s^{-1} for PEO 1M and 0.04-9.00 s^{-1} for PEO 600k, followed by a shear thinning behaviour. For PEO 600k 0.1 wt.%, the rheometer has problems measuring its viscosity for low shear rates. since it is a low viscosity liquid.

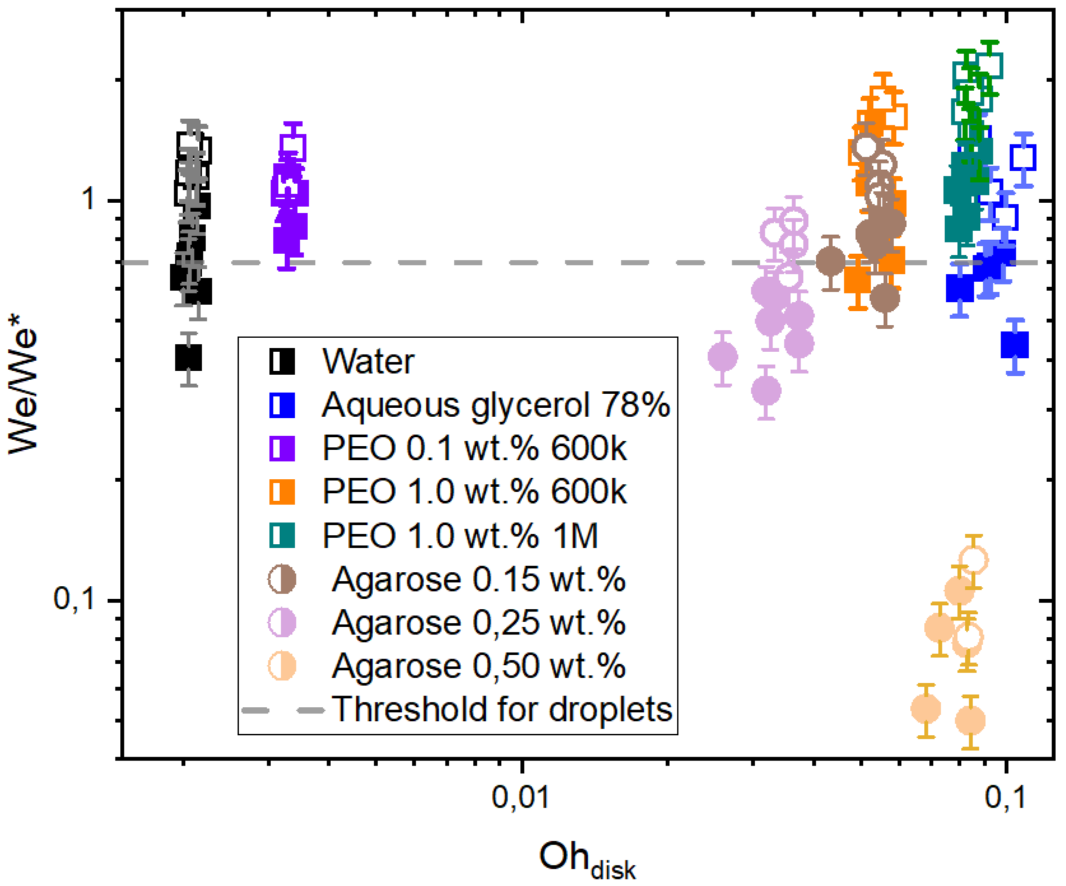

Fig S2. Phase diagram in log-log scale comparing the critical Weber number for traversing obtained experimentally (We) and from equation 11 (We∗). Each liquid and agarose gel are represented by their respective Oh number. Open symbols show cases where the jet traverses the droplet, while solid ones stand for the embedding case. The dashed line is We/We∗ = 0.7 the threshold found for droplets in[46]. The model is in good agreement with the experimental data for liquids and agarose 0.15 wt.%. In contrast, the threshold is underestimated for the agarose 0.25 wt.% and 0.5 wt.%. Where for agarose 0.5 wt.% the threshold is one order of magnitude lower.
